# From ‘aidland’ to ‘homeland’: what the lived experiences of Ukrainian crisis leaders indicate about humanitarian response

**DOI:** 10.1111/disa.70017

**Published:** 2025-09-19

**Authors:** Max Kelly, Maree Pardy, Dr Phoebe Downing

**Affiliations:** ^1^ School of Humanities and Social Sciences Deakin University Australia; ^2^ Centre for Humanitarian Leadership Deakin University Australia

**Keywords:** aidland, crisis, homeland, humanitarian, local, localisation, Ukraine

## Abstract

Reform of the international humanitarian system has stagnated, and commitment to localise power and resources has not eventuated. This research, specifically on the Ukraine humanitarian response, draws on anthropology's exploration of aidnographies, focusing on the oft‐overlooked role of aid workers; however, it offers an explicitly place‐based, grounded approach to Ukrainian crisis leaders' and responders' lived realities, decentring international ‘aidlanders’. We deploy the concept of ‘homeland’ as an explicit counterpoint to ‘aidland’ to portray the dilemmas, tensions, and contradictions of the international system from below. The study employs an actor‐oriented qualitative approach to examine the experiences of Ukrainian humanitarians. Semi‐structured interviews with 32 such individuals provide situated, contextual, time‐bound, and frequently deeply personal stories. Our respondents demonstrate the potential for international guests to adopt a ‘homeland’ sensibility, foregrounding a need to locate policy, procedures, actions, and decision‐making within an equitable, fair, and place‐based setting, responsive rather than controlling, and embedded in relevant contextual priorities, knowledge, and practices.

## 1 INTRODUCTION

Reform of the international humanitarian system (IHS) has stagnated. While important procedural and operational reforms have been implemented over several decades (Barnett, 2011), less effort has been made to examine critically the norms, principles, and institutions driving the system.

The latest reform agenda is a system‐wide commitment to localise aid. The localisation agenda, emerging from the 2016 World Humanitarian Summit, broadly calls for greater inclusion of ‘local’ actors, including an unfulfilled commitment to channel 25 per cent of humanitarian funding to them. Despite strong rhetorical support for localisation, progress has faltered, owing to systemic inertia and a structural inability to devolve power, authority, and resources (Kelly, Pardy, and McGlasson, 2024). Significant critique of localisation identifies its top‐down nature, reproducing rather than challenging existing power hierarchies, thus reinforcing the international–local binary, which embeds two classes of humanitarian actors: the international and the local (see, for example, Roepstorff, 2020; Khoury and Scott, 2024). This paper is situated therefore in the critical space of locally‐led crisis response, rather than localisation, acknowledging explicitly issues with the cleavage of the sector into local and international.

Local humanitarian actors have always been at the forefront of initial responses to crises; the IHS depends on the labour, knowledge, reach, and risk awareness of locals. This initial dependence, however, is often parsed by donors and humanitarian institutions as a temporary response prior to the arrival of international humanitarian professionals. Even when reliance on local actors is acknowledged, it is not accompanied by meaningful adjustments in power, authority, and resources. For instance, in September 2022, seven months after Russia's full‐scale invasion of Ukraine, an open letter^1^ to international donors and non‐governmental organisations (NGOs) from Ukrainian humanitarian civil society actors and organisations admonished the international community for its localisation failures. Despite many millions of dollars being raised internationally, Ukrainians stated that ‘we have failed to see resources coming our way’, calling for changes in the channelling and control of resources, and recognition of local actors' priorities and capabilities. The question posed by Ukrainians to the international community, ‘if not now, when?’, was an explicit expression of frustration about the system's inability to implement widely promised reforms.

This paper approaches the stagnation of the humanitarian system's reform through the concepts of *aidland* and *travelling rationalities*, both derived from anthropology's exploration of ‘aidnographies’. This approach counters conventional anthropological studies of development *outcomes* and recipients, to highlight the oft‐overlooked role of *aid workers* and how they perceive their work. Aidland is likened to a mysterious world akin to Alice's Wonderland composed of ‘places‐that‐are‐not‐places’ and lands whose ‘history [is] made from policy design’ (Mosse, 2011b, p. 2). Aidland is inhabited primarily by international aid and development professionals and experts, and rests on generalisable policy ideas produced by international organisations (Apthorpe, 2011). It is positioned as an apolitical, aterritorial realm detached from, and ‘standing above’, complex local contexts. In this surreal space, local actors are marginalised by a logic of international humanitarian exceptionalism. This aterritorial exceptionalism rests on what Craig and Porter (2006) call ‘travelling rationalities’, which prioritise the universal over the particular and the technical over the political (Mosse, 2011b, p. 3). These mobile rationalities stress processes over place, and categories over relations, transacting with the hard currency of expertise. Within aidland, the operational distinction between ‘local humanitarian labourers’ (Pascucci, 2019) and international humanitarian professionals congeals the systemic asymmetrical divide between local knowledge and international expertise.^2^

This paper therefore offers an explicitly place‐based, grounded approach to Ukrainian crisis leaders' lived realities. Our findings highlight the ill fit of these travelling rationalities, enacted through generalised and top‐down policies and practices in the context of Ukraine. In seeking to challenge this underpinning logic, the concept of *homeland* emerged as an explicit counterpoint to *aidland*. Homeland emphasises the particular over the universal, place and belonging over transcendent space, and a history produced through politics, cultures, and social worlds. It is thus a conceptual counter to aidland, portraying the felt dilemmas, tensions, and contradictions of the international system ‘from below’. The aspiration is to understand the lived experiences of crisis work in response to Russia's war on Ukraine, from the perspective of Pascucci's (2019) ‘humanitarian labourers’, who are abstracted from funding and control, which remains firmly in the hands of international donors and international NGOs (INGOs). We do not embed this paper in debates about humanitarian localisation, as the perceptions and experiences of the Ukrainian humanitarians with whom we spoke make clear that the ‘shift in power relations between actors, both in strategic decision making and control of resources’ (de Geoffroy and Grunewald, 2017, p. 2), that localisation promises, is not a feature of the Ukrainian response. The humanitarian system is not walking alongside Ukrainians in their homeland but imposing itself on them from elsewhere and above.

The research asks two principal questions:

- Who are the humanitarian labourers in Ukraine and what is their lived experience of leading the country's crisis response?
- What does this local lived experience indicate about the interface between local and international humanitarian structures, systems, and response?

After outlining the methodology, the paper turns to the Ukrainian humanitarian context. This is followed by a reflection on aidland, conceptually and practically, before we elaborate notions of homeland, as a geographical and political counterpoint to aidland (while also expressly reclaiming the idea of homeland from an overtly securitised notion embedded predominantly in domestic political contexts of the United States). The experiences of our Ukrainian crisis respondents are then presented, firstly as lived experience at the personal level, and then through consideration of their interactions with the IHS.

## 2 METHODOLOGY

This paper employs an actor‐oriented qualitative approach to examine the experiences of Ukrainian humanitarian crisis leaders and responders. We sought to understand actors who are frequently made invisible by agency‐ and systems‐centred research, to learn about their experiences, actions, and motivations within the humanitarian arena (Hilhorst and Jansen, 2012). This interpretivist research draws on data from 32 semi‐structured interviews with leaders of the Ukrainian response from the ‘escalation’ in 2022,^3^ 27 of whom were located within Ukraine, a further 4 were situated in Poland, and 1 was in France. All respondents were within NGOs. Of our respondents, 70 per cent were women and 14 per cent were working for INGOs. Fifty per cent of our male respondents worked for INGOs. Of those who shared their current role, eight were either founders, chief executive officers (CEOs), or senior leaders (director or deputy‐director level). Within Ukraine, five worked specifically in Zaporizhzhia, four in Kyiv, and one each in Chernihiv, Chernivtsi, Lviv, Khmelnytskyi, and Kharkiv; the rest were spread across multiple areas. Interviews were conducted between October 2023 and October 2024, and most were held in Ukrainian and translated. Participants were invited to share their stories, experiences, opinions, understandings, and roles in relation to serving as humanitarian leaders. Their stories are thus situated, contextual and time‐bound, foregrounding the subjective. Respondents were recruited from participants on a crisis leadership course, offered both within Ukraine and Poland, and through snowball sampling.^4^

Analysis comprised an iterative process of inductive thematic analysis. Berger (2015) highlights the importance of reflexivity and positionality in conceptualising research and analysing data; both are frequently absent in humanitarian research, which is widely presented as objective (Lokot, 2022). Emergent themes were identified independently by researchers and key themes compared to reveal potential bias. The participants are not representative of all humanitarians in Ukraine, and hence claims are partial, yet powerful. This research focuses entirely on the local response and does not engage with international humanitarian workers. We recognise that there are a wide range of individuals who inhabit ‘aidland’ and that there is a complex liminal space occupied by those who transition between the local and international realms. Yet, our iterative analysis highlighted the complex, personalised experiences of Ukrainian humanitarians. The narrative that emerged was strongly place‐based, framed in the political, economic, and cultural aspects of crisis response, revealing a deep disconnect with the rationalities of the IHS. Subsequent theorisation of this narrative engaged ‘aidland’ as a conceptual frame. Furthermore, as noted, given the ill fit of aidland for our humanitarian respondents, homeland emerged as a geographical and political counterpoint to it, for those for whom this humanitarian crisis is both local and personal.

## 3 HUMANITARIANISM IN UKRAINE: TOWARDS THE *SPACE* OF AIDLAND AND THE *PLACE* OF HOMELAND

A ‘master frame of exceptionalism’ (Saez and Bryant, 2023, p. 20) delineates dominant humanitarian paradigms. Crises are considered exceptional events requiring exceptional humanitarian interventions which sit above and beyond the crisis and its politics, in a space produced and provisioned by international humanitarian organisations. Such universalism underpins Craig and Porter's (2006) traveling rationalities. This classical humanitarianism is widely challenged (Barnett and Walker, 2015; Hilhorst, 2018; Adami, 2021) yet appears firmly entrenched in Ukraine. The Ukraine crisis and response are situated within a classical humanitarian paradigm insofar as it is a clear case of crisis triggered by direct conflict, a situation that calls for international aid and relief interventions, initially at the highest level (L3). Yet, Ukraine's robust civic response to the invasion provides a counter to this, both in supplying lifesaving relief to populations while unashamedly declaring opposition to Russia, alongside some form of recalibrating to a new normal, a position of both resistance and resilience (Hilhorst, 2018; Slim, 2022), which international humanitarians have failed to leverage properly, insisting instead that the local humanitarians fall into line with the IHS requirements for competence and accountability. Perhaps more crucially, as Noe and Lang (2023) emphasise, billions of dollars were channelled through the United Nations (UN) and INGOs, bypassing local organisations or turning them into agents of their foreign counterparts. By early 2023, the number of Ukrainian organisations working on crisis relief increased dramatically, performing 60 per cent of humanitarian activity in the country, yet receiving less than 1 per cent of funding (USD 3.9 billion in 2022). Furthermore, Cabot Venton, Noe, and Lang (2024) demonstrate that failure to accord Ukrainians greater control over the substantial international aid raised undercuts the speed and effectiveness of relief. This failure to ‘localise’ is arguably rooted in problems hinted at by Slim's (2022) notion of Ukrainian humanitarian labourers as not neutral but still humanitarian. Classic, principled humanitarianism remains steeped in the impossibility of being neutral in one's own country, reinforcing the centrality of the expatriate humanitarian expert.

There are existing analytical categories that focus on humanitarian action by civil society, operating external to or within the crisis space, but outside of the formal humanitarian aid architecture. In the development sphere, citizen initiatives have received attention but are focused on private endeavours from the Global North (Pollet et al., 2014; Haaland and Wallevik, 2017). Although there are some moves towards recognising the role of actors within a crisis context, such as those of Fechter and Schwittay (2019) and Brill (2020), a defining feature of these discourses is a spotlight on small‐scale, privately funded activity, and operations outside of formal structures (McCabe, Phillimore, and Mayblin, 2010; Fechter and Schwittay, 2019). Mcgeary (2024) highlights the often vague nature of reference to grassroots, and local, beyond the Inter‐Agency Standing Committee's definitions intended to clarify who is ‘local’ (IASC, 2018).

Our research foregrounds those leading and responding to the crisis in Ukraine, who operate across and above these framings, providing the on‐ground response. They are intentionally engaged directly with the IHS, as emphasised further in the data, but also are in tension with, and marginalised by, the same system. The framing of this research therefore reflects on a humanitarianism that is more than small scale, or external to that which is located in the transnational space, or gated compounds; it is also place‐based, and thus marked by a temporality associated with, but looking beyond, crisis, as experienced by those considered ‘local’. This question of who the authentic humanitarians are, haunts the international system and serves simultaneously to cement the real humanitarians as ‘disembedded from society’ (Hilhorst, 2018, p. 4). This disembeddedness is at the heart of aidland.

### 3.1 Encountering aidland

Scholarship on aidland emerged from anthropology's ethnographic turn to the spaces, places, and workers of international development. Mosse (2011a, p. vii) frames aidland as the ‘aggregate effect of expert thought and planning which is the virtual world of aid professionals’. His influential book, *Adventures in Aidland*, is a series of reflections on the experience of ‘living and working as part of the interconnected expert world of international development’ (Mosse, 2011a, p. vii). This work shifted the focus from traditional development literature that considers *whether* aid works, to *how* it works. Mosse's (2011b, p. 2) spotlight is on ‘regimes of truth and the internal dynamics of development’ that produce ‘professional identities, disciplines and the interrelation of policy ideas, institutions and networks of knowledge workers who serve the development industry’. Expertise is at the core of the discourse, how it is produced, perpetuated, and enacted. Extraordinary power and prestige are invested in global models and generalisable ideas that transcend specific realms.

Aidnographies, typically of expatriate staff, have gained increasing traction and resonance in studies of professional humanitarianism. For instance, Visser et al. (2016) analysed the work–life balance of humanitarian professionals, based on survey data from 142 expatriate staff with Médecins Sans Frontières. Strohmeier and Panter‐Brick (2022) highlight gendered experiences in high‐risk humanitarian spaces. Loquercio, Hammersley, and Emmens (2006) conducted more than 200 interviews with humanitarian staff from exclusively European, British, and American humanitarian agencies, to report on turnover in the humanitarian system. The third edition of the Antares Foundation's (2012) study on managing stress in humanitarian workers, initially published in 2006, construes ‘humanitarian workers’ primarily as expatriate field staff who are exposed to professional risks such as exhaustion, separation from family and friends, and employed on short‐term deployment contracts in other countries. This international cadre of global humanitarians, ‘deployed’ on a series of short‐term lifesaving ‘missions’, are the inhabitants of aidland, and the face of classical humanitarianism.

‘Aidland’ is further framed by concepts of volitional choice, whereby professionals willingly accept risk, danger, and personal sacrifice. In an influential study, for instance, Roth (2015) describes aid work as ‘edge‐work’, and a ‘form of voluntary risk‐taking’. This framing of preparedness as encountering ‘danger in Aidland’ *voluntarily* presents humanitarian work partly as a destination, and partly as an identity predicated on risk, stress, and ‘otherness’ (Strohmeier and Panter‐Brick, 2022). This risk is, however, mitigated for international workers through a ‘bunkerisation’ (Duffield, 2012) of aidland, an allegory in which foreign workers occupy an ‘aid archipelago’ that stands apart from the local environment. A resulting normalisation of a risk‐based segregation of foreign professionals from local populations cements different categories of staff (Weigand and Andersson, 2019) constructed through ‘humanitarian enclaves’ of compounds, cars, and hotels (Smirl, 2015). Access to these spaces is restricted, as local citizens (including humanitarian employees) lack the requisite capacity, expertise, and devotion to humanitarian principles, and are frequently perceived as potential security threats (Beerli, 2018). There is a clear paradox here: embedded risk requires this bunkerisation, but only of certain staff, presuming that local humanitarians are somehow less vulnerable to insecurity, or just not considered. Consequently, ‘aidland’ emerges from and reproduces the intractable binaries of the humanitarian system. Fechter (2012) posits the donor/beneficiary or aid giver and aid receiver binary as highly problematic, critiquing, in her study of international humanitarian workers in Cambodia, this ‘othering’ of the ‘world's poor’, or in this case, the ‘othering’ of the local humanitarians.

There are, of course, exceptions to this characterisation and oversight when it comes to ‘local’ humanitarians. Hietanen (2024) acknowledges the differential parental support available to ‘local workers’ as compared to their international colleagues. Nonetheless, the idea of considering local crisis leaders as more than beneficiaries, indeed as ‘real humanitarians’ in the Ukraine crisis, remains difficult to countenance in a humanitarian aidland.

Aidland therefore provides a key point of critique, from the perspective of those who are outside, or on the threshold of a humanitarian space encapsulated by the IHS. Furthermore, theorising of our data emphasised the need for a counter to both the allegorical device of aidland, and its accompanying suitcase of travelling rationalities. The Ukrainian humanitarian response is primarily enacted on the ground by Ukrainians. The crisis exists within an existential threat to Ukraine posed by the Russian invasion. Our participants framed their identity, motivations, and approaches within a clearly situated nationalism, knowledge, and adherence to ideals of Ukrainian victory, conceptually far removed from the aidland of Mosse (2011a) and others. In seeking to reconceptualise the space of our participants, as crisis leaders, as humanitarian responders, as Ukrainians, our research foregrounded notions of home, place, nation, and lived experience, providing the undeniable, but undervalued ‘expertise’ that holds no power in an IHS. These findings called for a ‘homeland’ focus based on the experiences, stories, and motivations of people who stepped into humanitarian work after their lives and livelihoods were disrupted by crisis.

### 3.2 From aidland to homeland

The concept of ‘homeland’ remains underexplored in humanitarian research. It has been utilised primarily by cultural anthropologists and political scientists, to investigate refugee‐led humanitarianism, notably, how displaced diasporic populations support their ‘ancestral homeland’ or country of origin (Malkki, 1996; Barnett, 2014; Watenpaugh, 2015; Olliff, 2022; McCallum Guiney, 2023).

The term is also central to studies of nationalism and ethnonationalism, and has resurfaced in politics and security studies, from which we intentionally distance ourselves. At its core, ‘homeland’ refers to one's country of origin or national identity. It is an inherently place‐based concept, but it is also deeply affective and emotional. As Smith (1986, p. 28) notes, it represents ‘an alleged and felt symbiosis between a certain piece of earth and “its” community’. The land produces the culture and its people, who in turn, reshape the land. Homeland, then, is more than geography, it is a sensibility that connects people to a collective sense of belonging, identity, and history. It may derive from ancient sentiments or be produced through modern nationalist ideologies, but it remains deeply felt (Kaiser, 2002). Geographers argue that no other term—whether culture, region, or area—captures people's attachment to place as well as ‘homeland’ does (Nostrand and Estaville, Jr., 1993; Smith and White, 2004).

We adopt the concept of ‘homeland’ in response to participants' account of their lived experience and the importance of, as well as their relationship with, the social, cultural, economic, and political context in providing a crisis response. Our research in Ukraine presents an opportunity to reconsider the paradigm of classical humanitarianism through a place‐based, radical shift, particularly as it relates to power dynamics between local actors and international institutions, from the standpoint of the former, a lens that is widely debated, yet poorly researched in action. There is an explicit conceptualisation of humanitarianism as something done to, or for others, and this othering of the donor/beneficiary, aid giver/aid receiver (Fechter, 2012) is at the heart of a homeland perspective that acknowledges humanitarians who are enmeshed in their own crisis, spatially, temporally, emotionally, culturally, and professionally. In putting forward a homeland lens, we challenge a discourse of humanitarianism that discounts the local, beyond an instrumental means of enacting a preordained programme. There have been some moves towards this sentiment in refugee‐led organisations (see, for example, Olliff, 2022), but little has taken place that challenges the complex space where aid receivers can be aid givers, where professionalism and expertise are recognised for their embeddedness rather than their exceptionalism.

We explicitly acknowledge that homeland is an emotive notion, broadly contaminated by overtly securitised terminology, embedded predominantly in US domestic political contexts. Its emotive power has been mobilised to unite populations against external threats. For instance, after the terrorist attacks on 11 September 2001, the US established the Department of Homeland Security, framing ‘homeland’ as an emotional attachment to the nation, grounded in shared history, and collective memory. Yet, as Ventura (2022) points out, in this regard, ‘homeland’ is subordinated to security agendas. Kaplan (2003) critiques this use of ‘homeland’, describing it as a tool to justify modern imperial power, drawing on emotion and historical imagery. This would be extremely problematic in a humanitarian setting where aid givers may be associated with nationalist or imperialist apparatuses, rather than people for whom attachment to the ‘homeland’ is resistance and struggle for liberation, as in Ukraine. Our approach explicitly rejects the conflation of ‘homeland’ and state security and nationalism. Instead, we seek to explore its deeper, relational meaning, particularly in the context of crisis and humanitarian response. This is emergent, but akin to Ventura's (2022) poststructuralist reading of homeland, framing it as a discursive construction, with space being relational.

Homeland, as a counter to aidland, shares an allegorical mission, to consider the world from a particular perspective, here, those who are of the space. Homeland presupposes that there are those of the homeland and ‘others’. Nostrand and Estaville, Jr. (1993) set out five basic tenets of a homeland: a people; a place; a sense of place; a control of place; and time. In Ukraine, the attachment to ‘homeland’ is resistance, and struggle for liberation, embodied through crisis response. The humanitarian system relegates a sense of place as irrelevant. A homeland perspective around control of place argues for the decentring of the IHS to a support role, rather than a controlling entity, and the temporal nature of the current crisis is a major focus of our respondents. Perhaps stretching the concept too far, international aid workers' connection to aidland, or to their homelands, can be drawn upon, using Safran's (1991) ‘teleology of return’ to consider how aidlanders may commit to integrating into their ‘host’ country.

The following section presents the experiences of our participants. Although ‘homeland as a counter to aidland’ is introduced above, it is in the analysis below that the notion emerged. We turn first to the question of ‘who are the humanitarian labourers in Ukraine and what is their lived experience of leading the country's crisis response?’

## 4 UKRAINIAN CRISIS LEADERS LIVED EXPERIENCES

### 4.1 ‘If not me, then who?’

The key themes of our participants' many, often deeply personal, stories are professional background, motivations, role in the crisis, personal and professional impact, and, crucially, how grounded each of these experiences was in being Ukrainian, living in, and responding to, a crisis on their own land, in their home, since the 2022 escalation.

Our participants are leading the crisis response in Ukraine. They are CEOs and founders of local NGOs and volunteer associations (UkrFLoc14, UkrFLoc15, UkrFLoc20, and UkrFLoc27), executive‐level leaders of regional government services providing social assistance for internally displaced Ukrainians (UkrFLoc1), and deputy country directors and programme managers of INGOs involved in the crisis response (UkrMInt25 and UkrMInt28), among a range of other positions. The context for our respondents was never straightforward and deeply connected to their embeddedness in the space. Some were both internally displaced persons (IDPs), while offering care to IDPs (UkrFInt24). Others (UkrMLoc5) housed IDPs in their own and neighbours' homes in the early days. Many had experienced personal trauma, loss of family members, displacement, fear, and uncertainty, and specifically in the context of providing a response, they had been exposed to risk, resulting in further trauma and burnout. The crisis came to them.

Beyond this intensely personal experience was the impact of the escalation on people's livelihoods; the imperative to earn a living while coping with trauma and the personal motivation to contribute or ‘help’ through volunteering. As the crisis evolved, also evident was growing professionalisation in terms of what ‘crisis response’ work means in Ukraine.

### 4.2 Becoming a humanitarian

Being a humanitarian and crisis leader in this setting is not a singular identity, not least in the professional lives of the study's participants. They are or have been accountants, business and marketing professionals, communications managers, lawyers, IT (information technology) managers, teachers, civil servants, bankers, journalists, and students. As one respondent said of her transition into helping Ukrainian refugees:

*I am a researcher, a PhD [Doctor of Philosophy] student. I worked in the sphere of environment protection before. I needed to stop since the escalation, because the regions where I need to do interviews and questionnaires there may be landmines* (UkrFInt18).

Alongside those transitioning into crisis response were a subset of respondents already working in Ukrainian civil society and social programming prior to the escalation, most as a result of Russian aggression since 2014, who reported pivoting their professional activities more towards humanitarian and crisis programming in 2022. For example, UkrFLoc1 held an executive role at a Centre for Social Services, primarily delivering psychological services. She described the impact of 24 February 2022^5^:

*We set up headquarters at a cinema from the city council. But in just a few days, we realised that . . . we also had to provide food for all these people. We were doing it in parallel*.

Respondents reflected on transferring their professional knowledge and skills into new roles in whatever way they could, such as: ‘I have quite an asset … an active professional past’ (UkrFLoc1). Still others reported feeling at a loss as to where to begin: ‘when the war started, no one had done anything before. We knew nothing about it. What is possible? So, is it possible to help charitable activities?’ (UkrFLoc12). PolMInt1 noted that ‘[a]fter 24 February … I came along to see what I could do to help … things to do grows in an avalanche accumulation’. As the conflict progressed and the ‘arena’ became more ordered following the chaos of the first few weeks, people and organisations evolved in their role, especially women. UkrFLoc11 expanded that ‘it's mostly only women who do the whole thing. … All men, well, most of them, are often at the front’.

There was little choice for these respondents, with the crisis unfolding in their lives, and in many cases in their homes and among their family members. Yet, they offer more than contextual knowledge, they transitioned significant expertise and experience to the crisis.

### 4.3 Volunteers and the transition to paid roles

A significant number of respondents, particularly the ‘accidental humanitarians’, worked in a voluntary capacity in the early phase, although several remained in a voluntary position on an ongoing basis (UkrFLoc26). UkrFInt24 provided insights into this evolution:

*We created a humanitarian centre for IDPs … as volunteers. We didn't have any funding. Firstly, we distributed food and hygiene products. Then, we started to cooperate with international or local organisations which had some resources to cover [the] basic needs of IDPs at that time. After that, there was a need to have long‐term projects and not just distribute goods*.

Those whose previous livelihoods and careers were no longer viable faced the twin concern of living in a conflict zone and maintaining a livelihood to feed and house themselves and their family members. UkrFLoc8 and UkrFLoc11 stated respectively:

*People who are working in this NGO, they are volunteers, so nobody gets anything from it. And all funds go to … hiring people to drive … paying for the warehouse where the stuff is stocked*.

*Volunteers. How could they not pay the salary? … Most of the people there are not rich*.

Within this group of volunteers, many reported moving into paid work or a ‘profession’ after a period of time. UkrMLoc23 volunteered for two months in Ivano‐Frankivsk, providing bulletproof vests and other products, prior to securing a paid position. Similarly, UkrFLoc22 reported:

*If helping in Sunday school can be called volunteering, then yes. We held various events and initiatives with children from the Sunday school for other geriatric nursing homes or orphanages. It lasted from 2013 till 2022. Later it became my profession*.

Many combined prior skills and experience with humanitarian experience gained on the ground to become a paid responder; however, transitioning to a paid role requires skills that are in demand by international systems. UkrMInt25, for instance, ran a successful logistics business prior to Russia's annexation of Crimea in 2014: ‘[I] was voluntary from 2014 till 2015. … I was helping not only IDPs, but also [the] Ukrainian army. I lost my business, which was flourishing prior to 2014. Unfortunately, it collapsed because of these hostilities’. He eventually got work at an international humanitarian organisation, primarily, he reports, because of his English‐language skills, rather than his logistics expertise. UkrMInt13 echoes this: ‘I realised that I had abilities such as English‐language skills, which are needed to help international organisations work’, highlighting the positioning of local staff in the system, as less professional, or suitable for lower‐level roles, even if they have relevant skills.

There is a strong reliance on volunteer labour in crises. Volunteers are globally feted for their selfless role in providing early‐stage humanitarian response (Baillie Smith et al., 2022), being a ‘critical bridge’ between beneficiaries and humanitarians (Alliance for Child Protection in Humanitarian Action, 2021) or ‘the buffer that fulfils the urgent needs left by incapable and ineffective failed States and International Organizations’ (Pereira Watts, 2016). The question of whether volunteers should be paid to secure their livelihoods is rarely raised. Pereira Watts (2016) claims that ‘[p]rivate companies have the budget and rapid mechanisms to create jobs, infrastructure and opportunities that emerge in the aftermath of the crisis and on the path towards peace and prosperity’.

There is limited but emergent research that addresses the issue of the financialisation of volunteering. Baillie Smith et al. (2022) argue for a livelihoods approach both for equity and to engage with the complexities of financialising volunteering, whereas Chadwick, Fadel, and Millora (2022) assert that volunteering can be used to gain entry into development work; however, there is a clear gap in the ‘non‐system’ within which ‘local’ volunteers, often driven by the collapse of their livelihood, and by a desire to assist, are recognised, supported, or enabled. A Ukrainian senior leader underlined this issue, with a positive example:

*At the beginning, we had the resources to help people, but we did not have the funding to support volunteers. We understand that volunteers can work there for a certain period, if they have the energy and motivation, as well as funding for their personal finances to ensure their lives and needs. So, after about three months, we felt that our volunteers were physically exhausted, emotionally exhausted, and financially depleted. So, when large international organisations came in, they gave stability to our employees because they met … their financial needs* (UkrMLoc5).

The rationale to continue assisting in the crisis response is, unsurprisingly, more complex than just being financial. Participants noted benefits with regard to their dignity, respect, and psychosocial well‐being (also countered by the psychosocial toll and burnout discussed elsewhere). For many respondents, volunteers or those employed, crisis response is not just a job, or salary. UkrFLoc27 made very clear that motivations are diverse:

*When we left our places, penniless, it was highly important for me that we could continue our volunteering activities. When you don't have your house, things … It was very important that international partners could find an opportunity to support our project, so that people could receive their salary and felt worthy. It's a very bad feeling … You had everything. Then you get up and you have nothing. It's about dignity*.

UkrMInt13 emphasised that war is stressful for everybody in the country, but ‘I realised that I could be useful’. UkrFInt18 commented that ‘[t]his volunteer work … helped me a lot because I needed to do something. Here I spent time with people, I helped them, and I didn't see what was happening in Ukraine. … It was hard to stay in my flat in Warsaw [Poland] and do nothing’.

For many participants, these motivations are complex and interrelated; PolFInt29 pointed out that ‘citizens … have become activists to help these people, because people were knocking on their doors asking for help’. UkrMInt10 remarked that it ‘was a new direction for me. But I realised that during the war it was necessary. … And with the war, it's almost … everyone's outlook, values, and attitudes have changed’. UkrFLoc20 observed that the ‘social capital I gain through this work is incomparable to … for example, to money or professional growth’.

Looking forward, many interviewees said that they would remain in the humanitarian or development sector professionally in the medium to long term. There was strong affirmation of both the current, ongoing crisis context, and the long road to recovery that lay ahead for the country and its citizens.

A strong sense could be gleaned from respondents' experiences that crisis work was not a choice as much as a necessity (lack of alternatives), a desire or moral imperative (to assist), *and* an opportunity (international funding for the humanitarian response necessitates staffing). The choice was not to go towards the crisis, but, being located within it, to live it, and to engage with it. Even among those for whom it offered a livelihood and a career, crisis work was presented as an imperative in a country that had dissolved into warfare. This was national survival, but there was also a sense of ‘if we don't, who will?’. Knowledge and expertise have evolved, presenting a place‐based rationality of how humanitarian and crisis response must be framed and enacted. Living and working in a crisis in one's homeland also lays bare the fallacy of a notion of volitional choice, and acceptance of the inherent risks of aid work, or at least a feeling of some protective mechanisms.

### 4.4 Personal and emotional impact

The personal toll of working in Ukraine's crisis response is profound, with one's country and city at war. ‘Nightmare’, ‘burnout’, ‘non‐stop’, and ‘pressure’ are some of the words that were used recurrently to describe the experiences of the interviewees. Many of the stories were framed in terms of specific incidents, and people.

Respondents conveyed a sense of the chaos, exhaustion, and adrenalin associated with providing an urgent response in the early days. ‘To be honest’, said one respondent of March 2022, ‘I don't even remember it very well, because my brain blocks everything negative’. She continued:

*I'm just trying to remember the faces of the people we saw then, and I can't remember them. … We had a lot of people pass through us. … I remember that March was very cold, and we had people who had left Mariupol in the winter. I still remember a woman in a bathrobe and slippers. I don't know how she got there. I mean, even if you just drive in a car like that, it's very cold* (UkrFLoc2).

The impacts were complex. UkrMInt25 recalled that ‘[b]efore 2014, I was much more tending to accept and much more empathic to people's sufferings, but now I feel there is a kind of blister somewhere on my heart’. UkrFLoc11 shared her frustration: ‘There are disabled people and old people, and I feel very sorry for them, and I want to help everyone. And you realise that you can't help everyone’. As the months wore on, the evolving emotional and psychological toll became increasingly evident. One senior manager working in regional social services close to the front reported: ‘I realised that after five months of non‐stop work at the headquarters, my employees were just burning out’ (UkrFLoc1). This was a key theme across respondents, for instance: ‘If you start to accept everything you see around you and take it very close to your heart, then you will very soon burn out professionally. You will need a psychiatrist support’ (UkrMInt25).

The ‘homeland’ perspective reveals the necessity of, and people's relative access to, psychological support. Much of the research around burnout and risk is positioned within ‘aidland’, those within the IHS, who in most cases can exit the arena. There is a clear need for resources for Ukrainian organisations to allow them to offer essential sustained and accessible psychological services to local staff and volunteers.

### 4.5 Solidarity, as Ukrainians, as locals

*This is definitely our country, this is our life, and for most international organisations it is just a job* (UkrFLoc20).

The aterritorial exceptionalism of international humanitarianism provides the moral high ground, the principled response, so widely touted. Our respondents, as Ukrainian humanitarians, could not easily be separated from their connection to Ukraine, their homeland. Evident in the descriptions of their work as humanitarians is the notion of belonging and serving ‘our country’, being responsive to ‘Ukrainian mentality’ (UkrMLoc5 and UkrFLoc9), not as neutral external observers, but as people whose lives were upended by the Russian invasion. This thinking recognises the homeland imperative, not just for Ukrainians but also for all humanitarians: ‘my programme manager from Türkiye came to support Ukraine, but when there was an earthquake in Türkiye in 2023 … she finished her response in Ukraine, because she understood that her family, her country, her people needed her’ (UkrFInt24).

When asked about the future, respondents demonstrated a keen awareness of the time limit on international support for response and recovery in Ukraine. The sense of belonging heightens the implications of these observations: the knowledge that when ‘they’ (the international actors) leave, ‘we’ (the Ukrainians) will remain to rebuild and reconstruct ‘our country’. One participant stated: ‘I think that our local NGOs will have enough work for another 10 years. International organisations will leave Ukraine sooner or later’ (UkrMInt28). UkrFInt24 added:

*I believe that this policy of nationalisation of employees in organisations is more significant for us. … Our international colleagues can support us in some way, mentor us, but they will not stay in our country, because they have their families in other countries*.

Even more complex is the question of de‐occupation and reconstruction, which are embedded in notions of victory for Ukraine. UkrFLoc2 stated: ‘There will be a lot of work to be done there [in the occupied areas]. What's the best way to put it? They need to be brought home. Because pro‐Russian sentiment was quite strong there even before the war’. Yet, these expressions of homeland are not simply affirmations of nationalism. They speak directly to the travelling rationalities of the international system, specifically access, trust, legitimacy, and the reach of the humanitarian response.

In traditional humanitarianism, the need for trust and legitimacy is used to justify principled response, primarily neutrality. However, as Hilhorst (2018) argues in her analysis of the role of ‘trust‐forgers’ in securing humanitarian access, the classical mono focus on humanitarian principles blinds the international system to other ways of securing access to crisis‐affected people, such as accountability, reliability, or even longstanding solidarity. Our Ukrainian humanitarians, in other words, are ‘trust‐forgers’, whose ability to work with the local population derives from the simple fact of belonging to it, a position that also renders them beyond classical definitions of neutral parties. As one respondent said of her sense of place as a Ukrainian and a humanitarian:

*We have to live here, we have to rebuild here, our place here is a priority. We have to make decisions … in favour of Ukraine. We are legitimate for the local population, for those who need this help. … We speak the same language, we understand the context and everything else … we have to be leaders in this* (UkrFLoc20).

From this perspective, belonging to a shared ‘homeland’ presents a complex challenge to neutrality. This is a core tension in the Ukrainian response, particularly among serving combatants and their families. Yet, the knowledge of those whose homeland is under attack and whose fellow citizens require assistance is proving essential for both an effective and efficient response. The aforementioned ‘open letter to international donors and NGOs who want to genuinely help Ukraine’ framed this issue clearly:

*We do not want to remain ‘neutral.’ The value of human life must come first, and supporting the needs of those on the front line can significantly reduce the amount of civilian aid needed and the number of casualties. Whilst we recognise that international organisations may want to be perceived as such, it should be up to local civil society in these circumstances to determine our own approaches and priorities*.

Lived experiences thus reveal explicit support for a locally‐led response, highlighting tensions with classical humanitarianism and associated principles, particularly neutrality.

This section raises significant issues with the widespread conceptualisation of abstracted experts, as the ‘risk takers’ and ‘aid givers’ of ‘aidland’. There is both a temporal and spatial aspect to our respondents' experiences that counter ‘aidland’. They were mostly compelled to move into crisis work when war upended their lives and careers. The context for them was intricately linked to conceptions of home. This is more than a job, and the choice to respond or not is heavily influenced by conflict on your doorstep, or in your neighbourhood and homeland. The sense that being in the crisis is a choice, or even a career, is bookended by highly complex decisions to stay, to engage, or to leave one's home. The risk and stress with which the IHS engages for aidlanders are absent, as locals have no access to Duffield's (2012) ‘bunker’. There is also a strong temporal aspect, with a longer‐term view on victory, recovery, and rebuilding. Our participants, as Ukrainian humanitarian leaders, and human beings of course, are firmly at odds with the rationalities, and principles, of a constructed reality, in which neutrality, impartiality, and independence can be realised by utilising an aterritorial and externalised logic. In fact, this very othering of the ‘local’ creates tensions that are borne by those on the ground. The next section explores the intersections of these personal experiences with the IHS and assesses how it supports and enables this response.

## 5 FROM THE PERSONAL TO THE SYSTEM: WHEN LOCAL AND INTERNATIONAL SYSTEMS COLLIDE

### 5.1 The need for international support

Moving from the personal to the system enables a ‘view from below’, decentring the IHS and its embedded inhabitants of ‘aidland’. A major aspect is the essential nature of resources that the IHS can bring to a context, relatively quickly. UkrMInt13 notes that ‘most humanitarian organisations in Ukraine operate thanks to international funds’. UkrFLoc9 shared that ‘[t]he lion's share of our work is done through cooperation with international organisations’, while UkrMInt25 stated that ‘funding by international organisations and international donors allows us to support [the] conflict‐affected population in Ukraine. … Without this funding our activities would not be possible’. The support and speed of funding in the initial phase was universally positive, providing a unique insight into where the IHS works exceptionally well. In the words of UkrMLoc5:

*They always had a small rapid‐response package of 50, maybe 70,000 dollars … for a quick reaction. And thank God that these were simple requirements for reporting, for tender proposals and everything else. That's why we were able to respond quickly for maybe some time*.

Beyond this initial phase, the humanitarian space became more formalised, invoking the travelling rationalities of global humanitarian aid. This became increasingly problematic, sparking significant advocacy for changes relevant to the local context and recognition of local actors. A locally‐led response is not sufficient in this setting, and our respondents valued, and actively sought to engage with, international resources and support. Our crisis leaders occupy the liminal space between aidland and homeland.

How the IHS was experienced is the focus of the following subsection, which addresses the ‘one‐size‐fits‐none’ and local–international binary themes.

### 5.2 One‐size‐fits‐none?

Major themes related to the ordering logic of the IHS replicate existing critiques, in terms of control and access to funding, donor requirements for reporting, and both the benefits of capacity strengthening and the challenge of being deemed to be lacking capacity, to meet the needs of the international system (see, for example, Jacobs et al., 2015; Roepstorff, 2020; UNOCHA, 2021; James, 2022). Three factors most specific to foregrounding a homeland perspective in relation to Ukraine are unpacked here:

- perceptions of partnerships;
- relevance of decisions to the context of Ukraine; and
- equity and risk between local and international staff.

As determined in the previous section, most of our respondents came to this space without knowledge or experience of the IHS. Each of the subsections below speak to their perceptions of, and interactions with, a system which they all portrayed very positively in terms of their initial interactions and as essential to providing resources.

#### 5.2.1 Perceptions of partnerships

*Working with INGOs can be like an abusive relationship, because we know we are dependent on them for funding. We can't continue offering our services without them … but we also need to stay strong to our values and not jeopardise the trust we have with our communities* (PolFlLoc29).

At a high level, the framing of partnerships raised some acute observations about the devaluing of the local. UkrFLoc2 recalled that ‘I came across this concept of a grassroots partner. … Well, I understand everything, but to me, it sounds a little bit humiliating?’. PolFlLoc29 felt that it was a double‐edged sword: ‘there is some strong benefits for local NGOs to partner with INGOs, but sometimes it's a barrier to local direction and leadership, reduces [the] flexibility of funding. We are inside the crisis’. The issue of language was vexatious to many. UkrFLoc11 noted that ‘[b]ecause I'm the only one who has to make reports … it's very hard. They are so meticulous, and they write in English, and well … I have a feeling when I make reports that they are not partners’. PolFInt3 explained that in ‘the beginning, for example, the introduction of the cluster system in Ukraine. Most of the general meetings were held in English. Only over time did they introduce semantic translation, which, of course, allowed more public figures and volunteer associations to join the meeting and understand what was happening’. Having an operational language other than Ukrainian both decentres the context and devalues locals who do not meet unreasonable demands for capacity in a foreign language.

Further entrenching the binary between local and international is a partnership that is deeply inequal. Partnerships were most often described in terms of ‘fulfilment partners’, or ‘implementing partners’, subservient in the relationship, where control rests with the external partner. This contributes to the second key factor, the context‐specific requirements of an effective response.

#### 5.2.2 Relevance of decisions to the context of Ukraine

The relevance of global humanitarian approaches and the experience of international staff can be misplaced. Some instances create frustrations, as highlighted by UkrMInt25:

*International experts come to Ukraine and they try to copy–paste the behaviour and programmes they have implemented in other parts of the world in our country. They don't even try to adjust these projects to the context of Ukraine, which is very sad to see*.

UkrFLoc2 highlighted some of her frustrations:

*Gulyaypole, where there is no electricity, no gas, no water, and people are cooking on the fire outside, risking their lives … I form sets of products that do not require significant effort to prepare. … So, they opened and ate the canned food. And when I take a soup from a Ukrainian organisation, she tells me, ‘okay’ … when I [go] to the international partner for consideration, he starts telling me about the approved one … in accordance with humanitarian standards*.

She added:

*In the same [area of] Gulyaypol … another organisation brought flour and pasta there. Excuse me, but how do you cook them? I mean, people perceive it as mockery*.

Other examples were beyond frustrating, producing potentially harmful situations. UkrFLoc27, based in the east of the country, shared:

*There were problems with reporting. Our international partners wanted us to prepare lists and … photos in 2022. But when you bring humanitarian aid to the occupied territory, it is dangerous for both the volunteers and the people who receive it. When international partners told us that they needed this kind of reporting, I refused to do it because the risk … of a person being tortured is high*.

Furthermore, the required response was said to be too embedded in the ordering logic of humanitarian funding. Different parts of Ukraine have wildly different contexts, with frequently fluid differentiation between development and humanitarian work. A late 2024 shift to a more recovery focused mindset clearly followed the ordering logic of humanitarian response, and was questioned. UkrFLoc26 recounted an organisational shift from relief, helping military personnel and IDPs, in 2014, to development work prior to 2022, and then to humanitarian aid based on need, and funding. UkrFLoc22 highlighted diverse needs, pointing out that ‘since 2022, new organisations have focused on providing the humanitarian aid, like food or other needs. They forgot about the development of civil society capacities, development of the country’. The differentiated needs of different parts of Ukraine were also raised in this context, particularly from east to west. ‘Procurement policies and international assumptions assumes 2024 is recovery and development stage on the INGO stage, but [the] everyday reality is that it is still crisis response and still inside the war’ (UkrFLoc14).

Responsiveness to context was demonstrated in the shift to more flexible cash programming, which was seen as a required change owing to early mistakes. UkrMInt25 noted:

*There is no need to provide free food now. This was the case in early 2022. Now we have markets working everywhere, except for the frontline territories that are being shelled very often now. Even there, people are finding working markets where they can buy whatever they need*.

The move to cash‐based programming addresses issues raised by UkrFLoc15:

*They [World Food Programme] almost killed economics in Zaporizhzhia region. They provided huge distributions of food for kids in a place where there were working stores and markets. So, people stopped [buying] rice, bread, and other stuff that they could buy in small shops in their villages*.

The imposition of ways of working, without recourse to local knowledge and realities, speaks to the clear tension between place‐based versus place‐neutral humanitarian interventions. Place‐based knowledge and experience can counter efforts that may be culturally, socially, or from a security perspective, inappropriate (or dangerous). Humanitarian funding also is limited, and the artificial boundaries between humanitarian aid and development through funding streams do not resonate with our respondents. They have a spatial and temporal view of the response that is far more nuanced. The findings of Cabot Venton, Noe, and Lang (2024) show that not only are place‐based interventions more relevant, but they translate directly into local programming that is 15.5 per cent more cost‐efficient than the activities of international intermediaries. The argument for a Ukrainian‐led response is therefore not only based on morals, but also efficiency and effectiveness.

Lastly, we turn to the demarcation of humanitarians as local or international and the felt implications of this narrative, specifically with regard to risk and equity.

#### 5.2.3 Equity and risk between local and international staff

Two final aspects were risk and equity of salary and opportunity. Respondents from multiple positions within the system, echoing a well‐understood issue (see, for example, Stoddard, Haver, and Czwarno, 2016; Roepstorff; 2020; Hamsik et al., 2022), highlighted the ethical implications of international actors engaging ‘locals’ to conduct high‐risk work,^6^ as articulated by UkrMInt25:

*International organisations … are not able to reach out to the frontline because of their security and safety restrictions. … They are signing local partnership agreements with local organisations, and they are asking these organisations: ‘Well, you are working in these areas, could you please help us with delivering … food items to that territory where we cannot go by ourselves because of our security restrictions?’. This is something that has not worked and will not work ever in the future, because this is an ethical problem*.

Burnout and exposure to trauma are intrinsic risks in humanitarian and crisis work. Risk discourse and planning is a major contemporary focus, yet research by Stoddard, Haver, and Czwarno (2016, p. 27) found that a stronger spotlight was placed on risk *of* local partners from a fiduciary perspective, than on the security risk *for* local partners. Ager et al. (2012) called for specific support for national staff, given both the increased risk and the gendered nature of burnout among locals.

Equity is the other core theme in relation to international and ‘local’ staff, repeatedly raised in aid discourse. UkrFLoc14 noted that a big problem in Ukraine is qualified project managers with experience of humanitarian response. With most men conscripted, and many leaving the country, UkrMInt21 argued that internationals have ‘taken the best specialists who gained invaluable experience from 2014’. The difference in salary and conditions among international organisations creates a dilemma for managers of local organisations: ‘If an opportunity opens for an individual in another organisation, it's not ethical to demand they stay’ (PoIMInt30). This leaves local organisations struggling to recruit and fill positions. UkrFLoc6 laid bare this dilemma regarding ensuring relevant staff: ‘It is quite difficult for local organisations to find specialists with experience, because international organisations set such high standards in terms of salaries that national organisations simply have nothing to compare with’; ‘Even drivers of international organisations receive much more than representatives of local organisations’. Building capacity is difficult in this context. Is it ethical to have to settle for a lower salary if your organisation is not international?

## 6 IF NOT HERE, THEN WHERE?

Given the perspectives and lived experiences of Ukrainian humanitarians, many of the well‐documented shortcomings in the international humanitarian system assume a sense of urgency. This research finds that the humanitarian response *could* have shifted its traveling rationalities. The response, as in many complex conflict cases, was primarily national, and strongly focused on civil society actors. Yet, funding and control have not been ceded in any meaningful way. The sense of solidarity and national identity that creates a firm foundation for crisis response is not leveraged or supported. The resources of the IHS are essential, but locals remain marginalised. These findings support Hilhorst's (2018, p. 7) argument that local institutions are depicted as more akin to ‘spoilers and causes of crises or in need of capacity building by international community’ and local people as ‘[v]ictims or cheats’. Participants' experiences foreground ‘locals’ as lacking capacity, and not principled enough to be considered ‘real’ humanitarians; instead, they are fulfilment partners, frontline labourers, required for the implementation of a humanitarian response, earning less than international colleagues, or in some instances not earning at all, in a context where livelihoods have been destroyed. They are subject to greater risk, and less reward. Aidland has superimposed itself on the crisis response in Ukraine, even while maintaining a safe distance in the implementation sphere. The expertise and professionalism of our respondents, the on‐ground crisis leaders, are subservient to those for whom the context is frequently entirely foreign. Our interviewees articulated their distance from, inability to access, and concerns with aidland. They further clearly highlighted their situatedness ‘within the crises’, their role as ‘trust forgers’, and their perceptions of the need to embed crisis response in the specific environment, their home.

The concept of ‘homeland’, and its inhabitants, is thus offered as a counterpoint to the narrative of ‘aidland’. A homeland perspective argues for centring people and spaces within which the crisis is unfolding, relegating the IHS to a support role. The nature of this is complex, in some cases, funding and resources are the clear requirements, in others, the experience of international staff is highly valued. Yet, our respondents demonstrated the need to locate policy, procedures, actions, and decision‐making within an equitable, fair, and place‐based context, responsive rather than controlling, and entrenched in relevant contextual priorities, knowledge, and settings.

The IHS is built on intractable binaries appertaining to local and international, brought about by the principled, risk‐taking, aid‐giving cadre of globalised ‘do no harm’ aidlanders. Such simplification obscures individual international aid workers' intentions and actions, but reflects a systemic corporatisation, securitisation, and hyper‐professionalisation of the field (Markland, 2025). This is expressed through ‘traveling rationalities’, the norms, values, and approaches created and maintained by travelling ‘experts’, whose principled claims are based on an identity that precludes geographical, political, or cultural relativity. Even if the rationalities and experiences gained elsewhere were completely appropriate to the context (which they are not), the IHS enters the home(land) of Ukrainians as an invited and, notably, highly welcome guest. It is where and how the ‘guest’^7^ engages the hosts that is shown to become problematic.

Drawing further on Ventura's (2022) essential elements for exploring homeland, our research underlines the *agency* of Ukrainian humanitarians as they resist Russia's invasion and invoking home and place while critiquing the actions of international humanitarian actors, demanding recognition from international systems. The *spatiality* of homeland does not necessarily align with the nation‐state but exists in relation to multiple political projects. It represents a concrete, evolving place, that is home to a community, yet always in flux. Ukraine as a nation under invasion is one aspect of spatiality, but the diverging experiences and needs of active conflict zones to less impacted areas are another. *Temporality* is also central: homeland is always in a state of becoming, shaped by both the past and the future. For Ukrainian humanitarians, the spatial dynamics between local actors and international humanitarians are deeply tied to the future. The focus of local actors on building a future, knowing all the while that international actors will eventually leave, shapes their humanitarian efforts and their frustrations.

This paper provides an epistemological alternative to an IHS that preserves the classical, predominantly Western idea(l) of humanitarian workers as voluntary ‘edge workers’ and professional ‘risk‐takers’ deployed to humanitarian crises elsewhere. It offers instead a view of humanitarians in the context of working in their homeland. Further research on ‘homeland’ as both a theory and a concept is needed. Yet, we suggest it as a compelling framework for understanding the relational dynamics we observed among Ukrainian humanitarians, their displaced co‐citizens, and the IHS.

We do not present homeland as a new paradigm for humanitarian response, nor as a revision of localisation. We offer it as a sensibility that the IHS would do well to cultivate, to reflect on the relationality between those in and of the homeland and those of humanitarian aidland, and to dismantle a local–international binary that prioritises an expertise that is external. Ukraine is not just a place of origin but a site of deeply felt belonging, agency, resistance, temporality, and transformation. We seek to position the concept of ‘homeland’ as a dynamic, relational, and evolving force that goes beyond geography, allowing for a critical reimagining of humanitarianism.

## CONFLICT OF INTEREST STATEMENT

The authors confirm that they have no conflicts of interest to declare.

## Data Availability

The data that support the findings of this study are available from the corresponding author upon reasonable request.

## Endnotes

^1^
For more information, see https://globalfundcommunityfoundations.org/news/an-open-letter-to-international-donors-and-ngos-who-want-to-genuinely-help-ukraine/ (last accessed on 20 August 2025).

^2^
Of course, the divide is not so clearcut in reality. People from within a crisis affected area, while perhaps deemed local, if working from within this context, may indeed be working for international organisations, and thus no longer ‘local’ under this framing. Equally, people who originate from outside a crisis context may have connections and experiences that render them more akin to a ‘local’. This is a complexity which is completely missed by humanitarian discourses that divide local and international as a founding principle.

^3^
In line with the language used by our participants, Russia's full‐scale invasion of Ukraine in 2022 is referred to as the escalation of the 2014 Russo‐Ukraine War, which saw the Russian occupation and annexation of Crimea, and support for war in the eastern Donbas region.

^4^
This research was conducted in compliance with the Australian Code for the Responsible Conduct of Research and received ethics approval from Deakin University (HAE‐022‐121). Informed consent was obtained both in writing (Ukrainian plain language statement and consent forms) and verbally from all participants before interviews were conducted.

^5^
The day of the full‐scale Russian invasion of Ukraine.

^6^
Risk in a humanitarian context is broader than security, but this was the predominant issue raised.

^7^
There is a complex correlation between the IHS and those working within it. We are referring in this paper to structural‐level issues; however, these are enacted through organisations, policies, and practices on the ground.
